# Supplementary material for: Alterations in Plasma Lipid Profile before and after Surgical Removal of Soft Tissue Sarcoma
Source: Metabolites. 2024 Apr 25;14(5):250. doi: 10.3390/metabo14050250 (PMC11123356; doi:10.3390/metabo14050250)
Supplement: Supplementary file 1 [file metabolites-14-00250-s001.zip › metabolites-2947583-supplementary.pdf]

Supplementary data S1. The patient information for plasma samples for soft tissue sarcoma

Individual	Gender	Age	Subtype	Location of primary tumor	Location of recurrence
1	M	62	Myxofibrosarcoma	Right forearm	None
2	F	76	Undifferentiated pleomorphic sarcoma	Right thigh	None
3	F	74	Dedifferentiated liposarcoma	Right thigh	None
4	F	51	Leiomyosarcoma	Right hip	Local
5	F	61	Leiomyosarcoma	Right thigh	Local
6	F	73	Undifferentiated pleomorphic sarcoma	Left hip	Local
7	M	42	Undifferentiated pleomorphic sarcoma	Right calf	None
8	F	61	Leiomyosarcoma	Left thigh	None
9	M	64	Leiomyosarcoma	Left thigh	Lung
10	M	65	Myxofibrosarcoma	Right upper arm	Lung
11	M	65	Myxofibrosarcoma	Right thigh	Local
12	M	56	Pleomorphic leiomyosarcoma	Left thigh	None
13	M	73	Myxofibrosarcoma	Right thigh	None
14	M	71	Myxoid liposarcoma	Left thigh	None
15	F	61	Malignant peripheral nerve sheath tumor	Left shoulder	Left thigh
16	F	75	Dedifferentiated liposarcoma	Left calf	None
17	M	46	Leiomyosarcoma	Right thigh	None
18	F	49	Well differentiated liposarcoma	Right hip	None
19	M	65	Pleomorphic liposarcoma	Right thigh	None
20	M	57	Angiosarcoma	Left hip	Local
21	F	57	Myxofibrosarcoma	Left thigh	None
22	M	60	Dedifferentiated liposarcoma	Retroperitoneaum	Local
23	F	42	Myxoid liposarcoma	Left thigh	Local
24	M	51	Leiomyosarcoma	Left thigh	Lung

Supplementary data S2. The collection time of post-surgery plasma, recurrence, and chemotherapy for individual patients.

Individual	Date of operation	Post-operative date of plasma collection	Date of recurrence	Pre- or post-operative chemotherapy	Distant metastasis
1	10/05/2019	16/05/2019	No recurrence	-	-
2	22/02/2019	07/03/2019	No recurrence	-	-
3	14/08/2019	21/08/2019	No recurrence	-	-
4	23/10/2019	10/11/2019	29/05/2020	-	-
5	16/09/2019	22/10/2019	20/04/2020	-	-
6	07/10/2020	14/10/2020	31/12/2020	-	+
7	11/11/2020	24/11/2020	No recurrence	+	-
8	21/05/2021	25/06/2021	No recurrence	+	-
9	29/09/2021	06/10/2021	16/02/2022	-	+
10	25/11/2020	02/12/2020	09/11/2021	-	+
11	04/09/2020	15/09/2020	11/10/2021	-	-
12	14/07/2021	23/07/2021	No recurrence	+	-
13	16/06/2021	25/06/2021	No recurrence	+	-
14	14/05/2021	24/05/2021	No recurrence	+	-
15	16/01/2019	21/01/2019	26/07/2019	-	+
16	08/05/2019	15/05/2019	No recurrence	-	-
17	14/08/2019	21/08/2019	No recurrence	-	-
18	05/11/2018	12/11/2018	No recurrence	-	-
19	30/03/2020	13/04/2020	No recurrence	+	-
20	04/03/2020	18/03/2020	06/12/2020	-	-
21	12/08/2019	21/08/2019	No recurrence	-	-
22	08/11/2019	23/12/2019	20/07/2021	-	-
23	26/04/2019	08/05/2019	06/09/2019	+	-
24	19/12/2018	26/12/2018	04/01/2019	-	+
